# Curcumin and Intestinal Inflammatory Diseases: Molecular Mechanisms of Protection

**DOI:** 10.3390/ijms20081912

**Published:** 2019-04-18

**Authors:** Kathryn Burge, Aarthi Gunasekaran, Jeffrey Eckert, Hala Chaaban

**Affiliations:** Department of Pediatrics, Division of Neonatology, University of Oklahoma Health Sciences Center, 1200 North Everett Drive, ETNP7504, Oklahoma City, OK 73104, USA; Kathryn-Burge@ouhsc.edu (K.B.); Aarthi-Gunasekaran@ouhsc.edu (A.G.); Jeffrey-Eckert@ouhsc.edu (J.E.)

**Keywords:** ulcerative colitis, Crohn’s disease, necrotizing enterocolitis, curcumin, inflammatory bowel disease

## Abstract

Intestinal inflammatory diseases, such as Crohn’s disease, ulcerative colitis, and necrotizing enterocolitis, are becoming increasingly prevalent. While knowledge of the pathogenesis of these related diseases is currently incomplete, each of these conditions is thought to involve a dysfunctional, or overstated, host immunological response to both bacteria and dietary antigens, resulting in unchecked intestinal inflammation and, often, alterations in the intestinal microbiome. This inflammation can result in an impaired intestinal barrier allowing for bacterial translocation, potentially resulting in systemic inflammation and, in severe cases, sepsis. Chronic inflammation of this nature, in the case of inflammatory bowel disease, can even spur cancer growth in the longer-term. Recent research has indicated certain natural products with anti-inflammatory properties, such as curcumin, can help tame the inflammation involved in intestinal inflammatory diseases, thus improving intestinal barrier function, and potentially, clinical outcomes. In this review, we explore the potential therapeutic properties of curcumin on intestinal inflammatory diseases, including its antimicrobial and immunomodulatory properties, as well as its potential to alter the intestinal microbiome. Curcumin may play a significant role in intestinal inflammatory disease treatment in the future, particularly as an adjuvant therapy.

## 1. Introduction

The incidence of intestinal inflammatory diseases, such as necrotizing enterocolitis (NEC), Crohn’s disease (CD), and ulcerative colitis (UC), is increasing worldwide. NEC is the most common gastrointestinal emergency affecting premature infants, and is associated with a high mortality rate and significant morbidity. The disease is multifactorial with, currently, poorly understood pathogenesis. A number of risk factors have been identified for developing the condition, including prematurity, hypoxic-ischemic injury, altered microbiome, and formula feeding [1]. NEC is largely characterized by intestinal inflammation and necrosis of the gut. To date, limited treatments for NEC are available, consisting of supportive treatment, surgical resection of damaged tissue, antibiotics, and rest of the bowels [1]. Many infants surviving NEC are subsequently subject to additional morbidity in the form of short-gut syndrome and neurodevelopmental impairments [2].

CD and UC, together referred to as inflammatory bowel disease (IBD), are chronic, relapsing inflammatory diseases with no cure and significant morbidity, most often affecting young adults [3]. Much like NEC, the etiology of IBD is, as yet, unexplained, but is thought to involve an overstimulation and excessive response of the intestinal mucosal immune system to resident luminal microorganisms [4]. In Crohn’s disease, inflammation is discontinuous and manifests as distinct granulomas, with inflammation often permeating transmurally and even affecting adjacent lymph nodes [5]. In contrast, ulcerative colitis, occasionally a milder condition, is characterized by continuous mucosal inflammation localized to the colon. Both CD and UC result in extensive epithelial damage. Treatment options for both diseases, including drugs such as cyclosporine, corticosteroids, 5-aminosalicylic acid (mesalamine), mercaptopurines, anti-tumor necrosis factor-alpha (TNF-α), and azathioprine, are costly, often involve significant side effects, and are limited in effectiveness and specificity [6].

The intestinal barrier is critical to health and is one of the most metabolically dynamic systems in the body. The intestines must constantly balance allowing molecules in (e.g., water, electrolytes, nutrients) while keeping inflammatory environmental antigens out [7]. Additionally, the intestinal barrier must manage the prevention of invading and translocating luminal bacteria, but also not become hyperreactive to these commensal or symbiotic microorganisms [1]. The intestinal barrier is composed of both an external physical and biochemical barrier and a complementary inner immunological barrier [7]. Wang et al. [8] have described the physical intestinal barrier as a four-layered system, where a strengthening in any one of these layers serves to strengthen the barrier as a whole. The four integral components of the physical barrier consist of (1) a lipopolysaccharide (LPS)-detoxifying alkaline phosphatase layer, (2) a physical mucin barrier that inhibits bacterial interaction with the intestine, (3) tight junctions, and (4) Paneth cell-secreted antimicrobial proteins (AMPs) [8].

The cells comprising the physical intestinal barrier are intestinal epithelial cells (IECs), a group encompassing mucus-secreting goblet cells, AMP-secreting Paneth cells, enteroendocrine cells, and absorptive enterocytes, among others [9,10]. IECs can not only sense microbes and microbial products, but they can respond by further reinforcement of their own physical barrier and coordination of the response by the intestinal immune system, becoming more or less tolerogenic as dictated by the intestinal luminal contents [11]. Commensal bacteria signal the development of tolerogenic dendritic cells (DCs) and macrophages by spurring IEC-derived production of retinoic acid (RA), transforming growth factor-beta (TGF-β), and thymic stromal lymphopoietin (TSLP) [11]. A tolerogenic immune population allows for the production of interleukin (IL)-10 and RA, both immunomodulatory compounds that can suppress pro-inflammatory cytokine production and promote the function of regulatory T cells [12]. Intestinal epithelial cells sense pathogenic microbes and microbial products via transmembrane pattern recognition receptors (PRRs). One class of PRRs expressed in IECs is toll-like receptors (TLRs). TLR4, in particular, a PRR recognizing LPS from gram-negative bacteria [13], is thought to play an important role in intestinal inflammatory diseases [4,14]. IECs can also respond to luminal bacteria by producing reactive oxygen species (ROS), which can both eliminate bacteria and signal for cell migration and epithelial repair [15].

The functional immunological barrier of the intestines lies largely underneath the physical barrier of IECs. The immune system of the intestine is composed of both innate and adaptive arms. As a newborn, adaptive immunity is less effective, so the infant relies primarily on innate immunity [16]. Innate immunity is comprised of primarily physical barriers (e.g., IEC mucus and AMP production), and a reactive component (e.g., resident and patrolling immune cells) [1]. Adaptive immunity is reliant upon antigen-presenting cells (APC), largely dendritic cells, which direct T and B cell differentiation and activation. Both goblet cells and specialized IECs, microfold cells (M cells), can present antigens to dendritic cells, priming the adaptive immune system [11]. From here, naïve T helper (Th) cells are differentiated into subsets (e.g., Th1 or Th2) with varying characteristics and cytokine profiles depending upon the local environment.

The pathogenesis of intestinal inflammatory diseases likely involves both IECs and intestinal immune cells. When the highly complex, bilayered intestinal barrier is either underdeveloped or disturbed, intestinal inflammatory diseases may result [17,18]. The breakdown of the intestinal barrier is most often attributed to overproduction of pro-inflammatory cytokines, such as TNF-α, IL-1β, and interferon-gamma (IFN-γ) [7], triggered by activation of the nuclear factor-kappaB (NF-κB) and activator protein 1/mitogen-activated protein kinase (AP-1/MAPK) pathways.

## 2. Intestinal Microbiome in Intestinal Inflammatory Diseases

### 2.1. Microbiome in NEC

The status of the microbiome in NEC has been widely investigated, but is inherently complex, involving frequent interindividual differences and temporal variability in microbial populations with development and progression of the disease and maturation of the intestine [19]. Intestinal microbes are known to play a role in NEC, as germ-free animal models are protected from development of the disease [20,21]. Preterm infants are recognized to harbor suppressed bacterial diversity, increased percentage of likely pathogenic flora, and reduced bacterial species compared to term infants [22,23]. The development of NEC has been correlated to relative increases in Proteobacteria phylum microbiota, while microbes in Firmicutes, Bacteroides, and Negativicutes are found in reduced proportions [24,25]. The role of microbial species diversity in NEC has been questioned, however, as meta-analyses have failed to find differences in either alpha or beta diversity indices when comparing babies developing NEC to healthy infants [26]. These changes in the NEC preterm infant microbiome may be innate to the maturity of the intestine, but may also be predicated on a number of risk factors for NEC development. For example, antibiotic usage predisposes preterm infants to the development of NEC [27,28], likely through increases in Proteobacteria and reductions in Firmicutes and Actinobacteria [26]. Interestingly, the microbial environment of the placenta and amniotic fluid, both previously thought to be sterile environments, clearly impacts the infant, as the meconium of babies developing NEC differs from those not developing the disease [29]. Additionally, higher intestinal luminal pH is associated with the development of NEC [30], and studies in babies prescribed H2 blockers demonstrated increases in Proteobacteria and a reduction in Firmicutes microbes [30,31], mimicking changes seen in NEC babies [25]. Finally, the role of breastfeeding in the development of NEC has been well established, and the microbiome of the milk appears to be highly individualized [32]. Breastmilk is known to reduce the incidence of NEC [33], potentially through oligosaccharide-associated increases in Bifidobacteria microbial growth [34].

Functional changes associated with shifts in the microbiome are increasingly recognized to be important, and will likely be a point of emphasis in future research. For example, short chain fatty acids (SCFAs), such as butyrate, are produced by gut bacteria and enhance the barrier function of the intestinal epithelium [35]. Host metabolism of butyrate leads to an environment less conducive to intestinal dysbiosis [36]. Intestinal microbial metabolites are also known to affect gut motility via indirect influences on serotonin production [37], a process which may influence the development or progression of NEC [35].

### 2.2. Microbiome in IBD

As with NEC, the development of IBD is thought to involve intestinal dysbiosis [38]. In IBD, a shift in the intestinal microbiota occurs, resulting in overall decreased diversity, reduced percentages of Firmicutes, and increased percentages of Actinobacteria and Proteobacteria [39]. In particular, pro-inflammatory *Escherichia* and *Fusobacterium* species are increased, while anti-inflammatory *Roseburia* and *Faecalibacterium* species are decreased [39]. Additionally, the microbial composition of IBD patients in remission compared to those with active disease differs, with those with active disease demonstrating higher levels of *Clostridium*, *Faecalibacterium*, and *Bifidobacterium* species [40]. Despite these general trends, human studies of microbial shifts in the context of IBD show very individualized differences [41].

Many of the risk factors for developing IBD-associated intestinal dysbiosis are similar to those of NEC, such as a lack of breastfeeding or caesarean instead of vaginal delivery [38]. However, the composition of the diet in IBD patients also appears to be highly relevant [42]. For example, diets low in fiber have been associated with an increase in the development of colitis, while high-fiber diets have been linked to protection from the disease [43]. Increased dietary fiber leads to the production of butyrate by commensal bacteria [44], known for its beneficial role in immunomodulation of regulatory T cells [45]. Both pre- and probiotics have also been studied in the context of IBD, but clinical trials have shown largely inconsistent results from these supplements [38].

## 3. Signal Transduction in Intestinal Inflammatory Diseases

### 3.1. NF-κB Signaling

**Figure 1:**
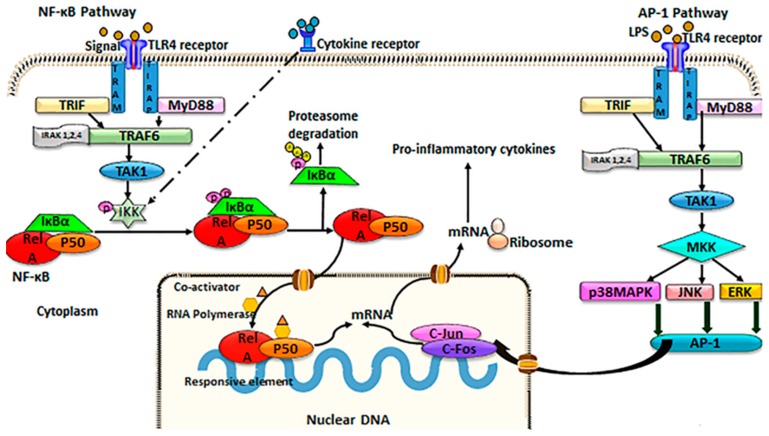
Schematic of TLR4/NF-κB/AP-1 signaling.

Both NF-κB and AP-1/MAPK pathways (Figure 1) are thought to play a role in intestinal inflammatory diseases [4,46,47,48]. NF-κB and AP-1 are ubiquitous transcription factors that bind DNA to regulate gene expression of inflammatory, differentiating, proliferative, and apoptotic genes. The NF-κB pathway can be stimulated via cytokine receptor ligands, PRRs, ROS, TNF receptor proteins, T cell receptors, and B cell receptors [49]. NF-κB is likely the dominant transcription factor involved in intestinal inflammatory diseases, and involves five subunits: p50, p65 (RelA), p52, cRel, and RelB [50,51,52]. These NF-κB components either homo- or heterodimerize to form active NF-κB [52]. In unstimulated cells, NF-κB resides in the cytoplasm, bound to inhibitory molecules of the IκB family that deem the proteins inactive [53]. Once stimulated, however, IκB proteins are degraded by the IκB kinase (IKK) complex [49]. The IKK complex includes the subunits IKKα and IKKβ, as well as the regulatory protein, NEMO (NF-κB essential modulator) [53]. IKK activation can be triggered by cytokines, microbial components, generalized cellular stress, and growth factors [49]. Following release into the cytoplasm, NF-κB proteins can translocate to the nucleus to bind to DNA promoters and initiate transcription of inflammatory genes, such as IL-1β, TNF-α, IL-12, inducible nitric oxide synthase (iNOS), cyclooxygenase-2 (COX-2), IL-23, and IL-6, as well as genes related to the function and activation of T cells [49,51,54]. Negative regulation of NF-κB signaling largely occurs through IκBα, which is able to translocate to the nucleus and negatively regulate NF-κB activation [55], interleukin-1 receptor-associated kinase-M (IRAK-M), a negative regulator of TLR signaling upstream of NF-κB, and through TNF receptor-associated factor 1 (TRAF1), which blocks the IKK complex [56].

Inflammation is a necessary defensive reaction of the host to both microbial infections and tissue damage, and is normally an acute and short-lived process. Dysregulated NF-κB signaling, however, can quickly lead to chronic inflammation and tissue damage. However, NF-κB plays a necessary role in healthy physiology. Interestingly, though NF-κB signaling occurs in both immune and IECs of the intestine [57,58], some evidence suggests NF-κB is protective in IECs, where it is necessary for the integrity of the epithelium, but inflammatory in intestinal myeloid cells. For example, studies in NEMO-deficient [59] and gastrointestinally infected [60] mice have indicated that an absence of NF-κB signaling in IECs leads to severe inflammation, indicating NF-κB can also play an anti-inflammatory role, depending on the context. Clearly, however, NF-κB is critical to IEC-driven lymphocyte development and host defense, particularly against pathogenic bacteria [55].

### 3.2. AP-1 Signaling

The AP-1 pathway, much like NF-κB, can be stimulated by LPS-activated TLR4 [61,62,63], growth factors [64], ROS [65], inflammatory cytokines [66], and generalized cellular stress. AP-1 consists of four DNA-binding families, the Fos, Jun, ATF/cyclic AMP-response element-binding (CREB), and Maf families, which homo- or heterodimerize [67,68]. The AP-1 pathway is also dependent on activation of MAPKs, which include extracellular signal-regulated kinases (ERK1/2), Jun N-terminal kinases (JNK), c-Fos-regulating kinases, and p38 [69,70]. The regulation of the AP-1 pathway is complex, with dimer composition, transcriptional and translational activity, and various protein interactions playing multiple roles [71]. When activated via pro-inflammatory cytokines and oxidative stress, as is most common in intestinal inflammatory diseases, the MAPK JNK translocates to the nucleus, phosphorylating c-Jun, activating transcription upon dimerization with c-Fos [71,72]. Target genes include pro-inflamatory mediators such as IL-1β, IL-6, IL-12, IL-23, iNOS, COX-2, and TNF-α [73,74,75], as well as matrix metalloproteinase 9 (MMP9) [76]. Adding to the complexity, though NF-κB and AP-1 are often differentiated as two separate signaling pathways, they are capable of modulating each other, with multiple overlapping downstream target genes [67]. For example, Mishra et al. [77] have demonstrated that c-Jun, a member of the DNA-binding families of AP-1, is necessary in NF-κB-dependent LPS signaling for transcription in macrophages.

The AP-1 pathway is active in both IECs [78] and immune cells [79] of the intestine. As with NF-κB, AP-1 signaling is believed to be necessary for healthy intestinal homeostasis and microbiota crosstalk [80]. For example, Wang et al. [81] have demonstrated that c-Jun is important in the resolution of intestinal wounds. However, dysregulated or unabated AP-1 signaling can lead to a number of physiological abnormalities, including excessive inflammation, and is believed to contribute to the development of intestinal inflammatory diseases [82,83,84,85].

### 3.3. TLR4 Induction

TLR4 is an upstream regulator of both NF-κB and AP-1, and its induction is critical to intestinal inflammatory diseases [14,86,87]. The TLR4 pathways aid in host immunity by allowing the host to distinguish between self and non-self molecules [6]. TLR4 signaling occurs in both IECs and intestinal immune cells [49], and is unique in that it operates, in both NF-κB and AP-1 signaling, through myeloid differentiation factor 88 (MyD88)-dependent and -independent mechanisms. In both MyD88-dependent and -independent mechanisms, ligand-induced dimerization of TLR4 is necessary to signal downstream [88]. In NF-κB, MyD88-dependent signaling, TLR4 ligand-binding recruits Toll/interleukin-1 receptor domain-containing adaptor protein (TIRAP), which then recruits MyD88 to the site. MyD88 interacts with and activates IRAK4, which then phosphorylates IRAK1. These IRAKs then detach from MyD88 and bind with TRAF6. TRAF6 activates transforming growth factor-beta-activated kinase 1 (TAK1), and TAK1, through TAK1-binding proteins, TAB1 and TAB2, activates IKK, initializing the NF-κB pathway [75]. In NF-κB, MyD88-independent signaling, TLR4 recruits the adaptor proteins TIR-domain-containing adaptor protein inducing interferon-β (TRIF) and translocation associated membrane protein (TRAM) [11,75]. Receptor-interacting protein (RIP1) associates with TRIF and TANK-binding kinase (TBK1) to form a signaling complex, which then regulates the downstream IκBα degradation [75]. RIP1 is also capable of signaling through a PI3K-Akt-dependent mechanism, negatively regulating mammalian target of rapamycin (mTOR) through NF-κB [89].

Alternatively, in AP-1, MyD88-dependent signaling, ligand-binding recruits TIRAP, and subsequently MyD88. Signaling progresses similar to that of NF-κB, MyD88-dependent (above) through the activation of TAK1. While in NF-κB signaling, TAK1 activates IKK, in AP-1 transduction, TAK1 activates MAPK family members (ERK1/2, JNK, and p38), leading to the activation and nuclear translocation of of AP-1 proteins (c-Fos, c-Jun, ETS domain-containing protein 2 (ELK-2), activating transcription factor 2 (ATF-2)) and gene transcription [77]. In AP-1, MyD88-independent signaling, ligand-binding recruits TRAM, and subsequently TRIF [75]. TRIF binds to TRAF6, leading to the activation of TAK1 [75]. From here, phosphorylation of MAPKs occurs similarly to the AP-1, MyD88-dependent pathway. TLR4 signaling is generally regulated by vascular endothelial growth factor-C (VEGF-C), nerve growth factor 1B (Nur-77), and selective androgen receptor modulators (SARMs), which negatively feedback to control this inflammatory pathway [75,90,91].

TLR4 signaling is important in that it primes the immune system, leading to the maturation of DCs and differentiation of Th1 and Th2 T cell subsets [4]. Additionally, TLR4 signaling promotes the differentiation of macrophages to an M1 phenotype, characterized by the production of pro-inflammatory cytokines [92]. Because TLR4 is upstream of both NF-κB and AP-1, however, denotes that a disproportionate TLR4 induction begets excessive inflammation [4].

## 4. Molecular Mechanisms of Injury in Intestinal Inflammatory Diseases

### 4.1. Pathogenesis of NEC

The pathogenesis of NEC is thought to involve the underdeveloped intestinal motility and barrier functions of the infant [93], an altered microbiome [94,95], and an immature, but hypersensitive, immune system [96]. Both innate and adaptive immune factors contribute to susceptibility to NEC, but the exact sequence of events in development of the disease is poorly understood.

In NEC, induction of the TLR4 pathway is not only implicated in the disease, but is thought to potentially be required for its development [87,97,98,99,100,101,102]. In normal physiology, IECs express very low levels of TLR4 [103]. However, in both mice and humans, prematurity is denoted by an unusually high expression of TLR4 [97,98,101,104,105,106]. Activation of TLR4 signaling in this environment not only leads to excessive inflammation, but also increased apoptosis of IECs, reduced migration and proliferation of IECs to replace those lost to apoptotic events, and the destruction of the intestinal epithelium [10]. The impaired intestinal barrier now allows the immature immune system greater and more frequent access to microbial antigens [107]. Dendritic cells residing in the intestine begin presenting antigens, and T cells, monocytes, and macrophages activate and initiate the production of a wealth of pro-inflammatory cytokines and chemokines [1,108]. This inflammatory cascade leads to recruitment of neutrophils, release of ROS, and further intestinal inflammation and necrosis [52]. Endothelial nitric oxide synthase (eNOS) is also reduced by TLR4 activation, potentially resulting in intestinal ischemia and necrosis [102,109]. A vicious cycle ensues, where inflammation begets more inflammation, overriding any attempts by the host of counterregulation. This inflammation spreads systemically, affecting organs as remote as the brain [2].

Evidence of the upregulation of TLR4/NF-κB/AP-1 signaling in NEC is robust. Preterm infants are likely developmentally predisposed to excessive NF-κB activation. In vitro and fetal cell explant studies of IECs have revealed that immature enterocytes display lower levels of the NF-κB-inhibiting IκBα compared to mature cells [110], resulting in elevated IL-8 production in response to LPS [111]. De Plaen et al. [52] demonstrated persistent NF-κB activation in intestinal epithelial cells in a rat model of NEC, while additional animal models have established that levels of NF-κB positively correlate with disease severity [14,112]. Managlia et al. [107] demonstrated that NF-κB activation occurs before the onset of intestinal injury, and that monocytes are differentiated into inflammatory intestinal macrophages during the very early stages of NEC via IKKβ. Fusunyan et al. [106] examined small intestinal histology from preterm infants with NEC and denoted both increased TLR4 and reduced IκB expression. Additionally, the neonatal intestine is also characterized by higher levels of c-Jun and c-Fos, important mediators in the AP-1 pathway [78]. Thus, the immature intestinal environment of the premature infant predisposes it to chronic inflammatory signaling.

A number of differences beyond the hyperinduction of TLR4/NF-κB/AP-1 have been noted between normal physiology and NEC, in both animal models and humans. IECs not only express PRRs, such as TLR4, but also present major histocompatibility class (MHC) I and II molecules [10], information presented to the adaptive immune system for future identification of foreign compounds. In NEC, infants generally show a lower expression of MHC II molecules [113], potentially allowing pathogenic bacteria to more easily translocate the intestinal epithelium. Goblet cells in the epithelium, characterized by protective mucin 2 (MUC2) production, are reduced in number and show decreased MUC2 production in both mice and humans [87,114,115,116].

The importance of neutrophils in the development of NEC is still unclear. Neutrophils, among the first cells recruited to the site of injury, release bactericidal compounds and ROS, and attract further immune cell recruitment [117]. Some animal studies have demonstrated a protective effect of neutrophil recruitment in NEC [118], while others have shown the oxidative metabolites from neutrophils may further degrade tissue impacted by NEC [119]. However, limited studies in humans have indicated that neutropenia is a significant risk factor for NEC [120], and immature human neutrophils show a reduced ability to phagocytize [121]. Meanwhile, macrophages in the immature intestine are hyperactive, demonstrating an increased sensitivity to microbial products [122,123,124], but the presence of TGF-β can suppress this immune activity [122]. Patients with NEC show increased tissue macrophage infiltration and suppressed levels of TGF-β2, an embryonic isoform [2,125]. Additionally, these macrophages may not be fully functional, as they, like immature neutrophils, show a reduced ability to phagocytize [121]. Dendritic cells, while not studied much in the context of NEC, may also contribute to the breakdown of the intestinal barrier [126].

Though adaptive immunity is less pronounced in neonates [16], T cells are still believed to contribute to NEC pathogenesis in a number of ways. For example, neonatal γδ intraepithelial lymphocytes (IELs), the first subset of intestinal T cells present during embryogenesis [127], produce higher levels of the cytokines IFN-γ and IL-10 compared to adult populations [128]. γδ IELs are thought to protect against bacterial invasion if mucosal injury occurs [129]. However, these γδ IELs are significantly less abundant in preterm infants with NEC compared to age-matched controls [127]. Tregs, T cells that regulate immune responses and promote tolerance, in both mice and humans, are found in lower levels in NEC infants compared to controls [98,130]. Th17 cells, a pro-inflammatory subset of T helper cells credited with tissue inflammation and destruction, are present in higher concentrations in the context of NEC [98]. The primary cytokine produced by Th17 cells, IL-17A, is believed to contribute significantly to NEC development by disrupting tight junctions, reducing IEC proliferation, and increasing IEC apoptosis [98]. Another subset of T helper cells, Th1, mediate, in large part, the cellular response to intracellular pathogens and microbial products. In NEC, there is some evidence these inflammatory mediators have a reduced ability to respond to pathogens and produce their signature cytokine, IFN-γ [131].

As expected given the upregulation of TLR4/NF-κB/AP-1 signaling in NEC, cytokine and pro-inflammatory ROS-associated enzyme levels drastically differ compared to age-matched controls [132]. For example, levels of inducible nitric oxide synthase (iNOS), an enzyme responsible for the production of nitric oxide (NO) and involved in inflammatory immune defense and oxidative tissue damage, are upregulated in NEC, both in tissue and serum [133]. TNF-α, a pro-inflammatory cytokine known to increase IL-1, provoke leukocyte migration, spur angiogenesis [134], and associated with shock [135], is increased in NEC [136,137,138,139]. TNF-α also increases levels of matrix metalloproteinases, such as MMP9, MMP12 [140,141], and MMP19 [142], destructive proteins which serve in the breakdown of the intestinal extracellular matrix [143]. Interestingly, the inhibitor of MMPs, tissue inhibitor of metalloproteinases (TIMPs), is also upregulated in NEC, likely indicating an attempt by the body at repair [144].

IL-1, a pro-inflammatory cytokine stimulated by TNF-α [134], is associated with leukocyte adhesion, macrophage and neutrophil activation [145], and the upregulation of IL-8 [146]. Levels of both IL-1α and IL-1β are upregulated in the NEC intestine [130,147]. Additionally, evidence suggests the neonatal intestine has enhanced sensitivity to IL-1β compared to more mature enterocytes [78]. IL-1 receptor antagonist (IL-1Ra), an anti-inflammatory protein competitively inhibiting both IL-1 isoforms, is also upregulated in NEC, though this upregulation is clearly not enough to counteract the rampant inflammation induced by IL-1 [148].

IL-6, a cytokine stimulated by a variety of pro-inflammatory cytokines including IL-1 and TNF-α, can act in both pro-inflammatory and anti-inflammatory means, and is an activator of lymphocytes in the adaptive immune system [149]. Levels of IL-6 are elevated in NEC [150]. Plasma IL-6, in particular, is significantly associated with higher NEC morbidity and mortality [151]. IL-8, a neutrophil and monocyte chemokine [152], is found in greater abundance in premature infants [111], but levels are upregulated further still in NEC, though often with a temporal delay [130,148]. IL-12, IL-18, and IFN-γ, which work simultaneously to increase inflammation via ROS [153,154], are upregulated in NEC [155,156,157]. Serum levels of IL-2 and IL-5 are also increased in the disease [158]. Finally, levels of IL-4 and IL-10, counterregulatory anti-inflammatory cytokines, are increased in NEC [130,148,158], while serum and tissue levels of the immune suppressor TGF-β are reduced [159]. The upregulation of IL-10, again, may demonstrate an attempt by the host at repair [1].

### 4.2. Pathogenesis of IBD

Whereas in NEC, pathogenesis of the disease is strongly predicated on prematurity, the development of IBD is thought to be dependent upon genetic susceptibility [160], lifestyle factors, such as diet and antibiotic use [161], intestinal barrier dysfunction [162], and, potentially, altered microbiome [163,164,165]. These factors, altogether, result in a heightened mucosal inflammatory response to luminal microbiota and breakdown of the intestinal barrier, likely through disruption of tight junctions [163,166,167,168]. Interestingly, increased intestinal permeability is often used clinically to predict relapse of Crohn’s disease, in particular [142,143], but intestinal permeability is, itself, not enough to initiate CD development, as first-degree relatives of CD patients, though asymptomatic, also demonstrate increased intestinal permeability [169,170,171,172]. As with NEC, the succession of events leading to development of IBD is not known.

Both the innate and adaptive immune systems contribute to IBD pathology. In a genetically susceptible individual, a small break in the intestinal epithelium, such as through bacterial translocation, activates the innate immune system, most likely through upregulated TLR4 activity. Activation of TLR4, and subsequently NF-κB and AP-1, promotes the enlistment of monocyte-derived macrophages, initializing production of pro-inflammatory cytokines, leukocyte-attracting chemokines, such as IL-8, monocyte chemoattractant protein 1 (MCP-1) and MCP-3, and macrophage inflammatory proteins (MIP) [163,173]. When inflammation is not constrained [174], APCs then enter mesenteric lymph nodes and drive T helper cell differentiation and the proliferation of macrophages, resulting in heightened sensitivity to luminal commensal bacteria [175]. Neutrophils then infiltrate damaged tissue and excessive pro-inflammatory cytokine release ensues, as well as the release of additional pro-inflammatory mediators, such as eicosanoids, MMPs, platelet-activating factor (PAF), reactive nitrogen species (RNS), and ROS [176,177,178,179,180,181]. MAPK activation in IECs spurs the upregulation of both COX-2 and iNOS, which can cause additional damage to the intestinal epithelium [182]. Furthermore, levels of vascular cell adhesion molecule 1 (VCAM-1), E-selectins, very late antigen 4 (VLA-4), macrophage 1 antigen (Mac-1), lymphocyte function-associated antigen 1 (LFA-1), and intercellular adhesion molecule 1 (ICAM-1) increase, attracting further recruitment and activation of lymphocytes [183]. Levels of counterregulatory mediators, meanwhile, such as TGF-β1 and IL-10, are reduced [184,185]. Thus, a vicious cycle of chronic inflammation ensues, resulting in the apoptosis of IECs [162], the prevention of apoptosis in, and accumulation of, T cells [5], and further compromise of intestinal barrier function. In the long-term, excessive signaling and inflammation resulting from microbial recognition by IECs has also been shown to drive colorectal cancer development in individuals suffering from IBD [186,187].

There is significant evidence of the upregulation of TLR4/NF-κB/AP-1 signaling in IBD. Levels of TLR4 are upregulated in the intestinal tissue of both humans [188] and animal models [189] of IBD. In both CD and UC, levels of tissue NF-κB are positively correlated with intestinal inflammation severity [51,190]. Intestinal mucosal macrophages of both UC and CD patients demonstrate increased levels of NF-κB, resulting in increased capacity for inflammatory TNF-α, IL-1 and IL-6 cytokine production [191]. In IBD, the p65 subunit of NF-κB is increased, particularly in Crohn’s disease [51,53]. Additionally, in an extensively utilized mouse model of IBD, trinitrobenzene sulfonic acid (TNBS)/ethanol-induced colitis, activation of the p65 subunit is a requisite step in the pathogenesis of the disease model [191]. AP-1 signaling has been deemed dysfunctional in IBD through increased JNK activity in both macrophages [192] and IECs [83,193]. Several studies have also indicated increased JNK activity in the inflamed mucosa of IBD patients [194].

IBD intestinal physiology vastly differs from that of healthy individuals, in both humans and animal models. For example, Paneth cells in IBD are known to release fewer AMPs, potentially allowing more bacteria to translocate the intestinal epithelium [195]. Recent research has also pointed to potential differences in autophagy in IBD, both of pathogens (xenophagy), as well as damaged mitochondria (mitophagy), leading to more microbial invasion and reduced clearance of damaged tissue [196]. Monocytes in IBD also show reduced MHC II expression, which has been shown to correlate with disease activity [197].

T cells contribute significantly to inflammatory bowel disease, and therapy aimed at T cell reduction has been shown to abrogate the disease [198]. In Crohn’s disease, in particular, T cells are extremely prevalent, and often form distinctive granulomas [5]. These T cells, primarily naïve, are recruited via the blood to the intestinal mucosa, largely through the production of adhesion molecules and pro-inflammatory cytokines [160]. An upregulation of IL-6 during IBD development leads to activation of the STAT3 pathway, preventing T cell apoptosis, and allowing for the abnormal accumulation of these cells in the intestine [199]. These T cells secrete large amounts of pro-inflammatory cytokines, permitting IBD disease progression.

In IBD, T helper cell profiles differ by disease. In CD, the profile of T helper cells is strongly skewed toward that of the Th1 and Th17 phenotypes [200,201], driven by LPS-associated IL-12 production [202]. Th1 cells, important in pathogen clearance, produce large amounts of IFN-γ, TNF-α, and IL-2 [5,203], and active CD lesions show high levels of these cytokines [204] and their associated T cells [205]. Th1 cells, much like naïve T cells in IBD, appear to be protected from apoptosis [206]. Th17 cells, the differentiation of which is spurred by IL-6 and TGF-β [207], are maintained via IL-12-associated release of IL-23 [208]. Th17 cells, native to the intestinal barrier and important in the elimination of extracellular pathogens, produce IL-17, an inflammatory cytokine aiding in neutrophil and monocyte recruitment [49,209]. Unlike in NEC, the role of IL-17 in IBD is not clear, as it plays a protective [210,211] and pathogenic role [212,213], depending upon the model. In UC, however, the T helper cell profile leans towards Th2, Th9, and Th17 phenotypes, producing large amounts of IL-5 and IL-13 [214]. Th2 cell differentiation is driven by IL-4, and when this pathway becomes dysregulated, upregulated Th2-associated production of IL-13 contributes to tissue destruction, as it induces apoptosis in IECs [215]. Th9 cells, meanwhile, driven simultaneously by IL-4 and TGF-β, produce IL-9, a pleotropic cytokine thought to impair the intestinal barrier function and exacerbate UC-associated tissue damage [216]. Additionally, the production of IL-21 may also play a role in IBD by driving both Th1 and Th17 responses [217].

T regulatory cells (Tregs) also play an important role in intestinal inflammatory disease pathogenesis, as they can inhibit effector T cells from functioning, promoting a more tolerogenic immune phenotype [218]. There is some indication the balance between effector T cells and Tregs is altered in intestinal inflammatory diseases [219], allowing for disease progression via inflammatory cytokine production and T cell activation positive feedback loops [220]. Further evidence of this imbalance is provided by studies denoting transfusions of Tregs into animal models of experimental colitis ameliorate the disease [221,222]. Treg function and Th17 differentiation is, in part, due to regulation by IL-18 [223,224], a cytokine found in higher levels in IBD patients [225].

Another group of lymphocytes thought to be important in IBD are the innate lymphoid cells, a population of lymphocytes which lack typical adaptive lymphocyte markers [226]. Patients with IBD, particularly CD, show significant mucosal infiltration of innate lymphoid cells, group 1 (ILC1), demarcated by production of IFN-γ and TNF [227,228], and mouse studies mirror this finding [229]. Type 3 innate lymphoid cells (ILC3s) also play a role, producing both IL-22 and IL-17. IL-22 is important in intestinal repair, and in mouse models, blocking this pathway leads directly to colitis [230,231]. IBD patients are known to have an increase in the IL-22 binding protein, an antagonist to IL-22 [232].

With increased TLR4/NF-κB/AP-1 signaling comes excessive production of inflammatory cytokines and oxidative molecules, some of which have already been discussed. Both CD and UC are characterized by increased synthesis of IL-1β, IL-6, IL-8, TNF-α, and IL-16, a T cell chemoattractant [51,160,233,234,235]. In the context of IBD, TNF-α and IL-1β are particularly important. TNF-α can activate resident tissue macrophages, spur further proinflammatory cytokine and oxidative inflammatory mediator release, as well as induce adhesion molecule expression, further driving leukocyte recruitment to areas of inflammation [160,233]. IL-1β, a pro-inflammatory cytokine associated with the innate immune response, has been found in high levels in the tissues of patients with IBD [236], as well as in monocytes from these patients [237]. In both humans and animal models, the balance of IL-1 and IL-1Ra plays a determinative role in IBD [238,239]. The IL-1Ra/IL-1 ratio is decreased in IBD, and this ratio correlates negatively with clinical severity of the disease [238,240]. In Crohn’s disease, the activity of inositol polyphosphate 5′-phosphatase D (SHIP), a negative regulator of IL-1β expression [241], is reduced, furthering the imbalance of IL-1Ra/IL-1. Both IL-1β and TNF-α can induce production of MMPs, further destroying the intestinal scaffolding [176,242]. MMP3, in particular, has been found in high levels in the tissues of IBD patients [243,244,245]. Overproduction of IL-6 is also thought to be important in IBD, where in mouse models, elevated IL-6 levels are directly involved in disease pathogenesis and can result in the abnormal accumulation of T cells in the intestine [199]. IFN-γ production, in the context of CD, is important in driving further production of IL-1, IL-6, and TNF-α [198]. Finally, the negative regulator of the immune-calming TGF-β, mothers against decapentaplegic homolog 7 (SMAD7), is increased in IBD [246], thereby blocking one counterregulatory measure to the excessive inflammation induced by these cytokines.

Pro-inflammatory cytokine release may result in tight junction disruption in IBD, further damaging the intestinal barrier [162]. For example, claudin-2, a pore-forming protein, is known to be upregulated in IBD, particularly in crypts where the protein is not normally present [235,247]. Additionally, the loss of tight junction strands and physical severing of these strands is associated with IBD-associated intestinal barrier defects [162]. Alterations in tight junctions, however, are thought to be a consequence of upregulated cytokine production, rather than a causative factor in IBD.

## 5. The Effects of Curcumin on Intestinal Inflammatory Diseases

Curcumin, the biologically active, hydrophobic, phenolic component of turmeric (*Curcuma longa*), is a natural product commonly utilized in Ayurdevic and traditional medicine, both topically and orally, for its potent effects on multiple body systems [248]. Four compounds, collectively termed curcuminoids and imparting a yellow color, are derived from turmeric, including curcumin, bisdemethoxycurcumin, demethoxycurcumin, and cyclocurcumin, with curcumin found in the highest concentration by weight [214,249]. Commercially purchased curcumin is often an impure mixture of approximately three-quarters curcumin, 17% demethoxycurcumin, 3% bisdemethyoxycurcumin, and little to no cyclocurcumin [248], and human curcumin clinical trial results have been complicated by the fact that multiple, heterogeneous mixtures of curcuminoids have been used in these studies [250]. Curcumin is characterized by the inclusion of two aromatic rings, and its phenolic hydrogens are believed to impart antioxidant activity to the molecule [214,248]. Curcumin, also known as diferuloylmethane, has been a popular supplement largely because of its affordability and safety, with no known toxic side effects in humans up to doses of 12 g/day [251].

### 5.1. Antibacterial and Microbiome Effects

Curcumin demonstrates a wide range of effects on the gastrointestinal system. In in vitro and in vivo models of *Helicobacter pylori* infection, curcumin inhibited bacterial growth on agar plates, and eradicated the bacteria from mice, respectively [252]. The bactericidal effect of curcumin appears to occur through an inhibition of bacterial cell division, resulting in the inappropriate assembly of the bacterial protofilament [253]. Further, Niamsa and Sittiwet [254] demonstrated the antimicrobial activity of curcumin against a number of commonly encountered pathogenic Gram-negative and Gram-positive bacteria.

Curcumin is also capable of regulating the gut microbiota, as a whole. Intestinal inflammatory diseases are defined, in part, by an altered, frequently pathogenic, microbiome [163,164,165,255]. In IBD, the microbiome is often enriched by a population of adherent invasive *E. coli* (AIEC), which can promote inflammation in the gut [256,257]. Studies investigating the effects of curcumin on the microbiome have attained different results depending upon the disease characteristics of the studied population. For example, in mice living in specific-pathogen-free conditions, curcumin supplementation decreased the microbial richness and diversity [258]. In a rat model of hepatic steatosis, curcumin administration reduced species richness and diversity, shifted the structure of the gut microbiota, and induced significant microbiota compositional changes compared to both high-fat diet and control groups, reversing the buildup of fat in the liver [259]. Importantly, curcumin favored the maintenance of short-chain fatty acid-producing bacteria, which are known to provide intestinal mucosal protection and inhibit intestinal inflammation [260,261].

In rats that have been ovariectomized, estrogen-deficiency-associated gut microbial shift is partially reversed by supplementation with curcumin [262]. Ohno et al. [263] showed, in a mouse model of colitis, an immunological and microbiological shift towards improved intestinal barrier function and reduced intestinal inflammation with nanoparticle curcumin supplementation. Curcumin administration in these mice significantly increased butyrate-producing microbiota, which are associated with colonic induction of Tregs, tolerance-promoting T cells [264,265]. McFadden et al. [266] utilized an IL-10-deficient model of murine colitis to demonstrate that curcumin supplementation prevented age-associated decreases in bacterial alpha diversity, increased bacterial richness, decreased *Coriobacterales*, increased *Lactobacillales*, and prevented development of colorectal cancer.

Studies on the effects of curcumin on the human gut microbiota are generally lacking, potentially due to the widely acknowledged absorption issues of the compound. Peterson et al. [250], in a pilot study, compared whole turmeric or curcumin extracts to placebo, and showed an increase in species and a trend toward increased alpha diversity with turmeric or curcumin supplementation. While individual responses to treatment varied, the patterns within the groups were very similar in both turmeric and curcumin, suggesting that curcumin, the most significant bioactive component of turmeric, was driving the observed changes. Interestingly, in subjects supplementing with turmeric or curcumin, the relative abundance of *Blautia* spp., believed to be the major metabolizers of curcumin [267], was reduced compared to controls [250]. While more complete studies on the effects of curcumin on the human microbiota are warranted, curcumin may be able to both simultaneously eradicate some pathogenic bacteria while globally shifting the composition of the intestinal microbiome.

### 5.2. Effects on Signal Transduction

Inflammation in intestinal inflammatory diseases is largely driven through upregulated TLR4/NF-κB/AP-1 signaling. Activation of TLR4 initiates an innate immune response and subsequent inflammation, in both NEC [14] and IBD [86]. Treatments abrogating TLR4-dependent signal transduction have been shown to lead to an amelioration of intestinal inflammatory disease [268]. Curcumin has been shown to inhibit both MyD88-dependent and -independent signaling mechanisms [88,269]. Additionally, curcumin can bind to myeloid differentiation protein 2 (MD-2), a protein bound to the extracellular TLR4 domain, thereby suppressing the innate immune response to LPS [270]. Additionally, should this initial inhibition not occur, curcumin can inhibit TLR4 signaling at a number of downstream steps, including TRAF6 and IRAK1, as well as through immune-modulating (e.g., MCP-1, MIP-2) and signaling-associated cytokine blockades [269,271]. In a Caco-2 model of the intestinal epithelium, treatment with curcumin resulted in diminished LPS-induced pro-inflammatory cytokine release and tight junction protein disruption [8], likely through a TLR4-dependent reduction in signaling. In TNBS-induced colitis rodent models, curcumin has been shown to ameliorate the disease through a reduction in TLR4 signal transduction [6,272].

**Figure 2:**
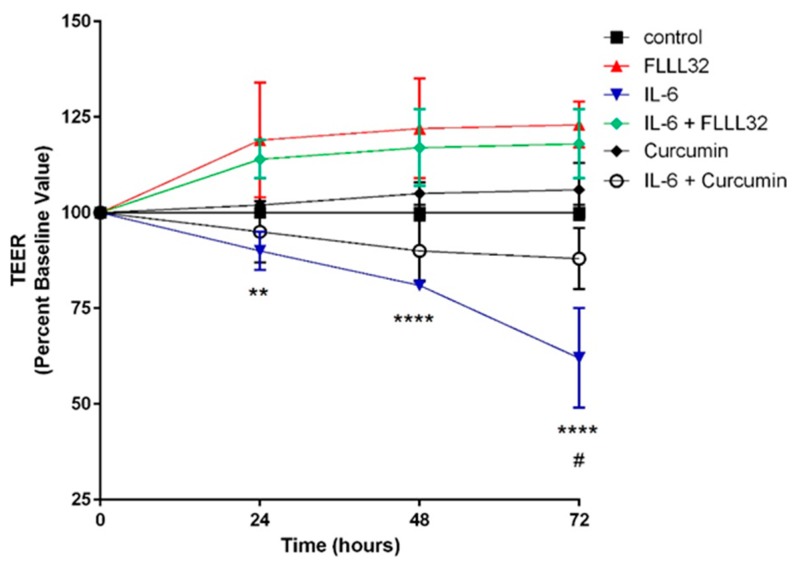
(Reprinted with permission from Dove Medical Press, Ltd.). Effect of FLLL32 and curcumin on IL-6-induced reduction of TEER in T84 monolayer. TEER value of T84 monolayers incubated with cell culture medium for 0–72 h in the presence of IL-6 (10 ng/mL) with FLLL32 (50 µM), curcumin (50 µM), or carrier (dimethyl sulfoxide) for 1 h in serum-free medium. ** *p* = 0.001 (IL-6 vs. IL-6 + FLLL32 at 24 h), **** *p* < 0.0001 (IL-6 vs. IL-6 + FLLL32 at 48 and 72 h), and # *p* = 0.003 (IL-6 vs. IL-6 + curcumin).

**Figure 3:**
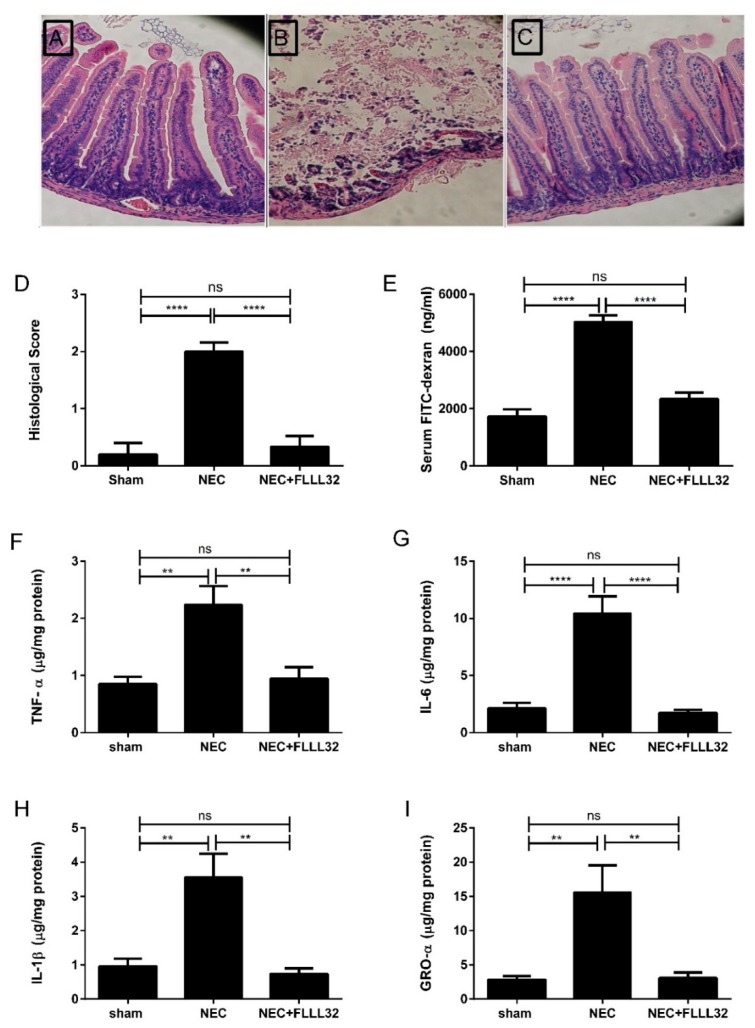
(Reprinted with permission from Dove Medical Press, Ltd.). FLLL32 attenuates intestinal inflammation and injury in DK NEC model. Representative H&E pictures from pups in the sham group (**A**), untreated NEC group (**B**), and NEC + FLLL32 group (**C**) (20× magnification). (**D**) Histological NEC scoring was obtained by two pathologists blinded to the groups (**** *p* < 0.0001). (**E**) FLLL32 preserved intestinal permeability in the NEC + FLLL32 group compared to the untreated group and control group (**** *p* < 0.0001). FLLL32 pretreatment reduced the levels of proinflammatory cytokines, TNF-α (**F**, *p* = 0.001), IL-6 (**G**, *p* < 0.001), IL-1β (**H**, *p* = 0.009), and GRO-α levels (**I**, *p* = 0.034) compared to pups in the untreated NEC group. Data are mean ± SEM. Results are representative of at least three separate experiments.

Eckert et al. [273] treated T84 intestinal epithelial monolayers with FLLL32, an analog of curcumin with greater solubility and potency. FLLL32 treatment reduced paracellular permeability associated with IL-6-induced inflammation, denoted as an alleviation of the IL-6-induced drop in transepithelial electrical resistance (TEER) (Figure 2). This same group, in a dithizone/*Klebsiella* Paneth cell ablation animal model of NEC, showed mouse pups treated with 25 mg/kg FLLL32 developed NEC less frequently, and at a significantly reduced severity, compared to pups with untreated NEC (Figure 3A–D; 20× magnification). Additionally, a fluorescein isothiocyanate (FITC)-dextran in vivo intestinal barrier assay demonstrated enhanced preservation of the intestinal barrier in FLLL32-treated animals compared to those with untreated NEC (Figure 3E). Finally, FLLL32 treatment decreased levels of the inflammatory cytokines IL-1β, IL-6, TNF-α, and growth-regulated oncogene-alpha (GRO-α) compared to levels in pups with untreated NEC, thereby inhibiting NEC-associated inflammation, likely through a TLR4/NF-κB-dependent reduction in signaling (Figure 3F–I).

Downstream of TLR4, signaling continues through either NF-κB or AP-1, both of which are upregulated in, and critical to, intestinal inflammatory diseases [4,46,47,48]. In both NEC and IBD, inhibition of NF-κB signaling has been shown to reduce injuries to the bowel [52,274,275]. Additionally, p38 MAPK inhibitors have established some success against colitis [192], including potentially in human IBD [276]. Curcumin inhibition of NF-κB activation appears to be through inhibition of IKKβ [277], thus reducing IκB kinase activity [278], and preventing NF-κB subunit movement to the nucleus. The mechanism of curcumin therapy in intestinal inflammatory diseases mimics that of steroids, blocking IκBα degradation in the cytoplasm and inhibiting nuclear translocation of the p65 subunit, in particular [51]. In AP-1 signaling, curcumin can inhibit MAPK [269], ERK1/2, JNK, and p38, both directly and indirectly, thereby limiting transcription of inflammatory target genes [279].

Numerous studies, in both animal models and humans, have documented curcumin inhibition of NF-κB and AP-1 signaling [280,281,282,283,284,285]. For example, in a variety of IEC lines, Jobin et al. [278] showed curcumin can inhibit NF-κB-binding to DNA, degradation of IκBα, translocation to the nucleus of RelA, serine phosphorylation of IκB, and activity of IKK. In HT29 IECs, in vitro treatment with curcumin inhibited TNF-α- and IL-1β-induced activation of p38 and JNK, while also inhibiting IκB degradation [286]. In a rat model of TNBS-colitis, curcumin treatment significantly reduced protein expression of MyD88 and NF-κB [6], prevented the degradation of IκB [287], and also alleviated symptoms of colitis via a reduction in p38 MAPK [288]. Sugimoto et al. [289] showed an amelioration of experimental TNBS-colitis in mice via a reduction in NF-κB activity. Additionally, curcumin pretreatment of mouse dendritic cells suppressed NF-κB translocation to the nucleus, as well as decreased phosphorylation of ERK, p38, and JNK [290].

### 5.3. Effects on Inflammation and Immunomodulation

The effects of curcumin on inflammation and immunomodulation are widely touted. Curcumin affects the function, differentiation, and maturation of a number of immune cells actively engaged in the pathogenesis or progression of intestinal inflammatory diseases. Dendritic cells treated with curcumin tend to promote the induction of intestinal T cells with a hyporesponsive phenotype, and these dendritic cells also demonstrate inhibited antigen presenting ability, leading to reduced stimulation of the adaptive immune system [291]. Curcumin-treated DCs also stimulate the differentiation of intestinal Tregs, and in a mouse model of colitis, these Tregs prevented the development of the disease [291]. Other studies have indicated that curcumin pretreatment suppresses LPS-induced NF-κB p65 translocation and MAPK phosphorylation in dendritic cells, leading to a reduction in inflammation [290]. Curcumin treatment of DCs reduces pro-inflammatory cytokine expression (IL-1, IL-6, TNF-α), and importantly that of IL-12, inhibiting the ability of these DCs to induce Th1-type responses [290]. Additionally, curcumin can reduce the dendritic cell expression of ICAM-1 (intercellular adhesion molecule-1) and CD11c, proteins related to both cellular adhesion and T cell activation [292], likely through an AP-1-dependent pathway. However, potentially the most important effect of curcumin on dendritic cells is to prevent their maturation via a suppression of indoleamine 2,3-dioxygenase (IDO), with an anti-inflammatory effect similar to that of corticosteroids [290,293].

Curcumin has been demonstrated to inhibit T cell-mediated immune functions playing a significant role in chronic intestinal inflammatory diseases [2,294], such as the ability to reduce the proliferative response of lymphocytes. This reduction in proliferation may occur due to both the antioxidant properties of curcumin, reducing ROS-related proliferation, and inhibition of ribonucleotide reductase and DNA polymerase activation, important in the cell cycle [294,295]. In addition, curcumin has been shown to reduce NF-κB-induced, T cell-initiated cytokine production [294], including the Th1-type cytokines, IL-2 and IFN-γ, further inhibiting lymphocyte proliferation [294,296]. In CD, Th1 cells predominate and are thought to drive much of the adaptive immune-related inflammation [200,201]. Curcumin can block production of the Th1 subset by suppressing macrophage production of IL-12, while also enhancing proliferation of the Th2 subclass [297,298], characterized by a more anti-inflammatory cytokine profile. For example, in a rat model of TNBS-induced colitis, curcumin at a dose of 30 mg/kg enhanced Th2 synthesis and suppressed Th1 proliferation, leading to a less inflammatory T helper profile [298]. Curcumin may also inhibit Th17 development, important in NEC [98], reducing production of the pro-inflammatory cytokines IL-6, IL-21, and IL-17 [299].

Dysregulation or hyperstimulation of the macrophage response [300], and alterations in function (e.g., decreased phagocytic ability in premature infant macrophages) [121] are critical in intestinal inflammatory diseases. Both monocytes and monocyte-derived macrophages in NEC infants exhibit an elevated expression of TLR4, TNF-α, and IL-6 compared to age-, sex-, and weight-matched controls, as well as lower levels of TGF-β1 [2]. Curcumin has been shown to inhibit TLR4 activation [6] and enhance production of TGF-β1, particularly in areas of active inflammation [301], such as the disrupted intestinal barrier. In rat macrophages, curcumin treatment at 30 mg/kg reduces the ability of cells to generate ROS and secrete lysosomal breakdown enzymes [302,303], leading to a potential reduction in mucosal inflammation. Curcumin can also inhibit NF-κB-induced macrophage and monocyte production of IL-12, IFN-γ, iNOS, MIP-2, IL-1β, IL-8, MCP-1, MIP-1α, and TNF-α [294,297,304,305]. Inhibition of IL-12 is particularly important in the context of adaptive immune cell differentiation and further progression of intestinal inflammation. In addition, several studies have indicated treatment with curcumin enhances the phagocytic activity of macrophages [298,306,307,308,309].

Intestinal inflammatory diseases are characterized by neutrophil recruitment and activation to the site(s) of injury, an early step providing a major source of ROS [310] for further mucosal and epithelial degradation. Curcumin is known to prevent neutrophil recruitment [288,311,312], largely accomplished through downregulation of NF-κB- and PI3K-Akt-induced chemotaxis [313,314], as well as a reduction in superoxide release [304]. Curcumin also inhibits neutrophils from aggregating, degranulating, and producing superoxide radicals [315]. In both B cells and natural killer cells, curcumin has been shown to enhance activity [295,316,317] or suppress activation [294,318], depending upon the dose and context [296]. Both B cells and natural killer cells have been identified as a potential general source of inflammation in the intestine [319], particularly in the context of microbial infection [320].

In addition to the effects on specific immune cells, curcumin alters the generalized production of cytokines across the entire intestinal immune system. Curcumin inhibits production of TNF-α [321], IFN-γ [322], IL-1 [323], IL-2 [294], IL-6 [290], and IL-8 [269], while elevating that of IL-10 [324] and TGF-β [325]. For instance, in both rat methotrexate-colitis and LPS-treated IEC-6 models, curcumin decreases levels of TNF-α and IL-1β, as well as increases levels of the anti-inflammatory cytokine, IL-10 [324]. In HT29 IECs, in vitro treatment with curcumin inhibited TNF-α- and IL-1β-induced IL-8 release [286]. In mice, curcumin has also been shown to suppress LPS-induced IL-12, IL-1β, IL-6, and TNF-α production [290].

Finally, COX-2, an inflammatory enzyme induced by NF-κB and AP-1 signaling, is an important mediator in prostaglandin synthesis. Levels of COX-2 are known to be upregulated in the context of intestinal inflammatory diseases [288,311]. In BV2 microglial cells, curcumin treatment abrogated COX-2 gene expression through reduction of both AP-1 and NF-κB signaling [326]. In addition to inhibiting the production of COX-2, curcumin can inhibit the receptors for COX-2 [311]. In a rat model of TNBS-induced colitis, curcumin reduced COX-2 expression, as well as the expression of several inflammatory cytokines, but increased levels of prostaglandin E_2_ (PGE_2_) [311]. In human colon epithelial cells [327], as well as HT-29 colonocytes [328], COX-2 has also been shown to be blocked by curcumin, likely through inhibition of NF-κB and IKK activity. Clinical trials with curcumin treatment have been largely successful, but the mechanisms of action have not been well-studied in these trials. For example, in quiescent ulcerative colitis patients, 2 g curcumin effectively maintainted remission [329]. In UC patients with mild-to-moderate disease, 3 g curcumin, in combination with the anti-inflammatory drug, mesalamine, induced remission in over 50% of the study population [330]. Both clinical trials likely depend on the anti-inflammatory effects of curcumin.

### 5.4. Antioxidant Effects

Curcumin is characterized by extensive antioxidant activity. Profligate oxidative stress plays a pathogenic role in intestinal inflammatory diseases [331,332,333], primarily through the breakdown of intact tight junctions [334,335,336]. Physiological levels of nitric oxide protect the intestinal mucosa [337,338], but the large amounts of NO released via iNOS, and potentially eNOS, during intestinal inflammatory disease progression can lead to tissue injury and necrosis [339,340]. Unabated generation of ROS and RNS can result in the peroxidation of membrane lipids, DNA damage, and the denaturing of cellular proteins [288]. In the intestinal mucosa, curcumin reduces levels of ROS, such as NO, superoxide anions, and malondialdehyde (MDA) [283].

During inflammatory events, iNOS produces nitric oxide in pathogenic amounts. In intestinal inflammatory diseases, chronic iNOS stimulation likely leads to the breakdown of the intestinal integrity due to this generation of RNS. In vitro experiments have demonstrated iNOS works in tandem with COX-2 through MAPK-dependent signaling, resulting in synergistic levels of inflammation and tissue destruction. In human tissues, increased levels of NO and iNOS expression have been demonstrated in intestinal inflammatory diseases [156,341]. iNOS expression appears to be a critical step in experimental colitis models, as iNOS-deficient mice do not develop the disease [341]. iNOS production is inhibited by curcumin [300,305]. In vitro studies have demonstrated curcumin can scavenge excess NO effectively [342,343], and in rat colitis models, curcumin can downregulate iNOS expression and decrease tissue levels of nitrite [288,321,344]. Finally, in a mouse model of TNBS-colitis, curcumin inhibits production of iNOS and peroxidation of lipids via reducing the Th1 cytokine response, leading to diminished tissue damage [283].

Myeloperoxidase (MPO), a component of monocyte and neutrophil granules, produces high levels of ROS. MPO is often used clinically as a marker of neutrophil infiltration into the intestinal mucosa [345]. Curcumin has been shown to decrease intestinal inflammatory disease-associated MPO activity in animal models of colitis, thereby limiting oxidative tissue damage [288,298,311]. For example, in an immune-mediated model of mouse colitis, Mouzaoui et al. [321] demonstrated curcumin is capable of reducing neutrophil intestinal infiltration, thereby reducing MPO activity, as well as returning NO levels to baseline via inhibition of iNOS and reduced inflammatory cell infiltration. In a rat model of TNBS-colitis, treatment with curcumin significantly reduced activity of MPO [6]. Additionally, in a rat methotrexate-colitis model, curcumin decreased intestinal MPO and increased levels of free radical-scavenging superoxide dismutase (SOD) [324]. These effects appeared to occur via a mitogen-activated protein kinase phosphatase 1 (MKP1)-induced reduction in p38 phosphorylation, as well as inhibition of IκB cytoplasmic degradation [324].

Matrix metalloproteinases (MMPs) are enzymes required in the degradation of the extracellular matrix. MMPs are upregulated in intestinal inflammatory diseases largely due to pro-inflammatory cytokine production. Research in humans suffering from IBD has established the intestinal epithelium overexpresses levels of MMP1, MMP3, MMP7, MMP9, MMP10, and MMP12 [346]. Infiltrating leukocytes and vascular endothelial cells were determined to be the source of MMP7 and MMP13 [347], while macrophages produced MMP8, MMP9, and MMP10 [348]. Neutrophils were largely responsible for MMP9 [348,349]. Matrix metalloproteinases have not been well studied in NEC; however, MMP3 is known to be upregulated in the disease [143]. Curcumin is known to inhibit the large majority of these matrix metalloproteinases, though their inhibition has not been extensively investigated in the context of intestinal inflammatory diseases. For example, in human umbilical vein endothelial cells (HUVEC), curcumin inhibits the expression of MMP9 [350], while, in cartilage explants, curcumin reduces MMP3 [351]. In human fibroblasts, curcumin downregulates MMP1 and MMP3 expression through a MAPK-dependent pathway [352], and in HT29 cells, curcumin reduced production of MMP7 [353].

Finally, curcumin can upregulate phase II enzymes related to the metabolism and detoxification of xenobiotics [354], as well as additional antioxidant proteins, such as nuclear factor (erythroid-derived 2)-related factor (Nrf2) [354], a transcription factor functioning as a master regulator of antioxidant proteins, and heme oxygenase-1 (HO-1) [355,356], a redox-sensitive, stress-induced protein capable of degrading heme to iron, biliverdin, and carbon monoxide (CO) [357]. In an in vitro model of rat hepatic stellate cells, Liu et al. [358] showed curcumin upregulates the nuclear translocation of Nrf2, thereby protecting the cells from oxidative stress. HO-1 can be induced by a variety of ROS, including H_2_O_2_. In a Caco-2 model of the intestinal epithelium, Wang et al. [359] indicated curcumin reduced the oxidative stress and cytotoxicity induced by H_2_O_2_ production. Additionally, curcumin was protective against H_2_O_2_-induced tight junction disruption, and its associated increase in paracellular permeability [359].

## 6. Conclusions

In this review, we discussed the potential protective effects of curcumin on intestinal inflammatory diseases. IBD and NEC are characterized by hyperstimulation of the immune system to luminal bacteria and dietary antigens, resulting in rampant intestinal inflammation. This inflammation impairs the functioning of the intestinal barrier, allowing for increased bacterial translocation, systemic inflammation, and in very severe cases, sepsis. Recent research has focused on the effects of natural anti-inflammatories, such as curcumin, on intestinal inflammatory diseases, largely due to their safety profile and affordability. Curcumin is characterized by beneficial effects on the microbiome, antimicrobial properties, inhibition of TLR4/NF-κB/AP-1 signal transduction, changes in cytokine profiles, and alterations to immune cell maturation and differentiation. The culmination of the vast number of effects of curcumin on the intestinal epithelium and immune system is to strengthen the intestinal barrier through a reduction in bacterial translocation and inflammation. While curcumin looks promising in the treatment of intestinal inflammatory diseases, further controlled clinical trials are needed.

## Abbreviations

AIEC	adherent invasive *E. coli*
AMP	antimicrobial protein
APC	antigen-presenting cell
AP-1	activator protein 1
ATF-2	activating transcription factor 2
CD	Crohn’s disease
CO	carbon monoxide
COX-2	cyclooxygenase-2
CREB	ATF/cyclic AMP-response element-binding
DC	dendritic cell
eNOS	endothelial nitric oxide synthase
ELK-2	ETS domain-containing protein 2
ERK	extracellular signal-regulated kinases
FITC	fluorescein isothiocyanate
GRO-α	growth-regulated oncogene-alpha
H&E	hematoxylin and eosin
HO-1	heme oxygenase-1
HUVEC	human umbilical vein endothelial cells
IBD	inflammatory bowel disease
ICAM	intercellular adhesion molecule
IDO	indoleamine 2,3-dioxygenase
IEC	intestinal epithelial cell
IEL	intraepithelial lymphocyte
IFN-γ	interferon-gamma
IKK	IκB kinase
IL	interleukin
ILC	innate lymphoid cell
IL-1Ra	IL-1 receptor antagonist
iNOS	inducible nitric oxide synthase
IRAK-M	interleukin-1 receptor-associated kinase-M
JNK	jun N-terminal kinases
LFA	lymphocyte function-associated antigen
LPS	lipopolysaccharide
M	microfold
Mac-1	macrophage 1 antigen
MAPK	mitogen-activated protein kinase
MCP	monocyte chemoattractant protein
MD	myeloid differentiation protein
MDA	malondialdehyde
MDPI	Multidisciplinary Digital Publishing Institute
MHC	major histocompatibility class
MIP	macrophage inflammatory protein
MKP-1	mitogen-activated protein kinase phosphatase 1
MMP	matrix metalloproteinase
MPO	myeloperoxidase
mTOR	mammalian target of rapamycin
MyD88	myeloid differentiation factor 88
MUC	mucin
NEC	necrotizing enterocolitis
NEMO	NF-κB essential modulator
NF-κB	nuclear factor-kappaB
NO	nitric oxide
Nrf2	nuclear factor (erythroid-derived 2)-related factor
ns	not significant
Nur-77	nerve growth factor 1B
PAF	platelet-activating factor
PGE_2_	prostaglandin E_2_
PRR	pattern-recognition receptor
RA	retinoic acid
RIP	receptor-interacting protein
RNS	reactive nitrogen species
ROS	reactive oxygen species
SARM	selective androgen receptor modulator
SCFA	short chain fatty acid
SEM	standard error of mean
SHIP	inositol polyphosphate 5′-phosphatase D
SMAD7	mothers against decapentaplegic homolog 7
SOD	superoxide dismutase
TAB	TAK1-binding
TAK	transforming growth factor-beta-activated kinase
TBK	TANK-binding kinase
TEER	transepithelial electrical resistance
TGF-β	transforming growth factor-beta
Th	T helper
TIMP	tissue inhibitor of metalloproteinase
TIRAP	toll/interleukin-1 receptor domain-containing adaptor protein
TLR	toll-like receptor
TNBS	trinitrobenzene sulfonic acid
TNF-α	tumor necrosis factor-alpha
TRAF	TNF receptor-associated factor
TRAM	translocation associated membrane
Treg	regulatory T cell
TRIF	TIR-domain-containing adaptor protein inducing interferon-β
TSLP	thymic stromal lymphopoietin
UC	ulcerative colitis
VCAM	vascular cell adhesion molecule
VEGF-C	vascular endothelial growth factor-C
VLA	very late antigen
